# Gender Identities in Organized Sports—Athletes' Experiences and Organizational Strategies of Inclusion

**DOI:** 10.3389/fsoc.2020.578213

**Published:** 2020-10-29

**Authors:** Birgit Braumüller, Tobias Menzel, Ilse Hartmann-Tews

**Affiliations:** Institute of Sociology and Gender Studies, German Sport University Cologne, Cologne, Germany

**Keywords:** LGBT+, gender identity, sexual orientation, social exclusion, minority stress, discrimination, inclusive strategies, sports organizations

## Abstract

In relation to conceptualizing sports, beliefs about sex binary and male hegemony are dominant. To match these assumptions and provide level playing fields, sport systems are based on sex-segregation. Thus, people who do not fit into or reject fitting into sex categories are hindered from participating in sports, particularly organized sports. Studies on social exclusion of gender-identity minorities in sports mainly adopt a qualitative approach and focus on Anglophone countries. This research is the first to provide a comprehensive picture of the experiences of LGBT+ athletes in organized sports settings in Europe and is based on a quantitative online survey (*n* = 2,282). The current paper draws special attention to differences between cisgender and non-cisgender athletes (including transgender men, transgender women, non-binary, and non-identifying individuals). Besides athletes' experiences, organizational strategies of inclusion, derived from qualitative interviews with stakeholders from sport systems in five European countries (Germany, Scotland, Austria, Italy, and Hungary) are examined. Theoretically anchored in Cunningham's (2012) multilevel model for understanding the experiences of LGBT+ individuals and Meyer's (2003) minority stress model, the paper aims to (1) analyze the assessment of transnegativity and (2) examine negative experiences (prevalence, forms, perpetrators) of LGBT+ athletes from organized sport contexts in Europe; and (3) discuss inclusive strategies in sports organizations in Europe. Data reveal that transnegativity is perceived as a major problem in European sports, and non-cisgender athletes are the most vulnerable group, suffering particularly from structural discrimination. The implementation of inclusive strategies for non-cisgender athletes is perceived as a complex and essential task, but the sports organizations in the five countries differ substantially in terms of the status of implementation.

## Introduction

Recent legal decisions inside and outside the sport system, as well as cases of prominent sport stars (e.g., boxer Patricio Manuel, triathlete Chris Mosier, or runner Caster Semenya), highlight the issue of transgender and intersex athletes' inclusion in sports and especially in competitive structures of sports. In discussions on this topic, transgender athletes are often blamed for challenging the binary sex-segregated system of elite and recreational sports, having unfair physical advantages, and calling fairness and the level playing field of sports into question. Conversely, sports organizations are accused of implementing discriminatory policies, of adhering to binary sex-segregation instead of considering gender identity or other competitive categories, and of systematically excluding transgender athletes and preventing the positive effects of sport on transgender individuals (e.g., in Sykes, 2006; Karkazis et al., 2012; Gleaves and Lehrbach, 2016; Jones et al., 2017b; Semerjian, 2019). These arguments about systemic bias are supported by evidence of individual attitudes toward and personal experiences of transgender individuals in sports (e.g., Smith et al., 2012a,b; Jones et al., 2017b; Cunningham and Pickett, 2018; Devís-Devís et al., 2018).

For some time now, transgender issues have been discussed in Anglophone research, mostly under the LGBT(IQ) umbrella (Kavoura and Kokkonen, 2020). This approach of lumping transgender athletes together with other sexual minorities has been questioned, because these groups face different challenges and prejudices in sports (Lucas-Carr and Krane, 2011; Cunningham and Pickett, 2018; Semerjian, 2019). In the European context, transgender inclusion in sports, as well as experiences of and barriers to transgender sports participation, are rarely quantitatively examined on a broader level. For example, quantitative research in the Netherlands has focused more on sexuality and sexual orientation (Elling et al., 2003; Elling and Janssens, 2009), while research in Scotland has considered both gender identity and sexual orientation (Smith et al., 2012a,b). Without reference to sports and sports participation, the European Union Agency for Fundamental Rights (FRA – European Union Agency for Fundamental Rights, 2014) found that almost half of the transgender respondents in a study reported experiences of discrimination and harassment in the year prior to the study and that transgender individuals are often exposed to physical attacks. Furthermore, in the year prior to the study, 38% of transgender respondents were discriminated against in places and situations other than workplaces, amongst which sports clubs were included (FRA – European Union Agency for Fundamental Rights, 2014).

Although transnegativity is perceived as a problem in sports (Smith et al., 2012a), the European research corpus is truly deficient. This was the initial starting point for the current research, which is the first to provide a comprehensive picture of the situation and to present experiences of LGBT+ people undertaking sports in Europe^1^. The current paper focuses on two entangled topics: (1) situations and experiences of cis- and non-cisgender individuals^2^ within LGBT+ communities in countries of the European Union, based on a quantitative online survey; and (2) organizational strategies and perceived barriers and facilitators for LGBT+ inclusion, based on qualitative interviews with stakeholders from sports organizations in five European countries.

## Sex Binary (in Sports)

Sex binary is based on opposite categories and ignores the spectrum of sex characteristics (Griffin, 2012). According to such categorization, a person is either a man/male or a woman/female, or in the words of Krane and Symons (2014, p. 122) “to be male and masculine is not to be female and feminine” and vice versa. Whilst sex refers to biological aspects of the body (e.g., hormones, chromosomes, physiology), gender is a socially and culturally constructed reference for being masculine or feminine according to bodies and behaviors (Enke, 2012; Griffin, 2012; Krane et al., 2012).

The term *transgender* describes an incongruence between sex and gender: namely, the sex assigned at birth does not fit the inner feelings of gender identity (Krane et al., 2012), defined as “one's sense of one's self as a gendered person” (Enke, 2012, p. 12). Following Enke (2012, p. 19), transgender “may include a gender identity that differs from the sex assigned at birth; a gender expression that differs from that conventionally expected of people according to their bodily sex; and/or a desire for alteration of the body's sex/gender characteristics.” Transgender serves as a category that includes transgender males and females, non-binary persons (i.e., individuals with gender identities that are neither exclusively female nor male), and sometimes also non-identifying persons (i.e., individuals who do not identity as male, female, or transgender). Gender expression focuses on how “people express, wear, enact, and perform gender through behavior, mannerism, clothing, speech, physicality, and selective body modification” (Enke, 2012, p. 18). Therefore, gender expression can take many forms, ranging from styling and self-presentation, to the changing of names and pronouns, to hormonal and medical treatments (Griffin, 2012). Persons who do not conform to the assumed gender roles and expressions are known as gender non-conforming individuals.

Sports and physical activities are a way to express one's gender (identity) by engaging in what is considered typically masculine, feminine, or gender-neutral sports; playing sports in specific ways; or meeting/rejecting sports-related gendered role expectations. Perceptions of adequate sports engagement and acceptable sporting behavior are strongly orientated toward the sex binary and differentiate between accepted and valued behavior for boys/men or for girls/women (Lucas-Carr and Krane, 2012). Girls/women still face several challenges in the domain of sport, in which masculine characteristics (i.e., playing hard, being aggressive and assertive) are promoted, and the masculine body is the norm, reflecting a “hegemonic notion of athleticism as a masculine trait” (Griffin, 2012, p. 101). The normative presumption of male dominance in sport and the physical advantages of men substantiate sex-segregation in the sport system, which was theoretically established to provide equal opportunities and maintain fair competitions (Griffin, 2012; Lucas-Carr and Krane, 2012).

The sport system is based on the sex binary and assumes that athletes can be unambiguously separated into the rigid categories of biological sex assigned at birth. To participate in competitive sports, individuals have to “align themselves as female or male and join the corresponding team,” which is an unconscious decision for the majority of athletes (Krane et al., 2012, p. 15). Thus, transgender individuals are strongly challenged by having to align to the sex binary and adhere to sports policies that mostly require a match of biological sex and gender identity (Griffin, 2012). One of these challenges is because adaption, the movement from assigned sex at birth to gender identity, happens at different levels and stages, ranging from personal and social recognition to hormonal/medical treatment to sex reassignment (Enke, 2012).

As sex-segregation is based on the assumption of male bodies' superior physicality, transgender athletes are perceived as challenging gender boundaries (Griffin, 2012) and contradicting the level playing field in sports (Lucas-Carr and Krane, 2012). In particular, female transgender athletes, who transitioned from male sex assigned at birth to female gender identity, are accused of having unfair advantages over so-called natural women. Griffin (2012) and Semerjian (2019) question this and emphasize that gaining competitive advantages over others is a fundamental feature of sports, and advantages can occur in different forms, among which physicality is only one of many advantages (e.g., social, economic, environmental, and psychological advantages).

## Theoretical Framework on Exclusion, Discrimination, and Minority Stress

Social theorists have developed different perspectives and discourses on social inclusion and exclusion, drawing on social inequality (Durkheim, Bourdieu) or systems theory (Luhmann, Parsons) (Kronauer, 2010a). Based on Weber's category of social closure, the inequality discourse deals with social exclusion as missed opportunities for social participation and an intensification of social inequality (Kronauer, 2010b). Following Durkheim's work, social inclusion is based on the achievement of social cohesion and organic solidarity, whereas Marshall sketches social inclusion as linked to the granting of social rights, among others, that provide equal access to institutions and an appropriate standard of living (Kronauer, 2010b). Access to and participation in organized sport contexts can be understood as a social right, that should be granted to every individual independent of social status, ethnicity, sexual orientation, or gender identity.

Apart from that, sports and sport engagement are seen as a means for social inclusion or at least as able to contribute to inclusive processes. According to Bailey (2005, p. 76), sports are theoretically assumed to contribute to inclusion on different dimensions: bringing people together from different backgrounds (“spatial”); enabling people to share a sense of belonging (“relational”); providing opportunities to develop competencies and capabilities (“functional”); and increasing relevant social networks and community cohesion (“power”). However, just as the existence of these dimensions can lead to inclusion, their absence can foster exclusionary processes and discriminatory experiences in sports.

For understanding discrimination based on gender identity, Cunningham's (2012) multilevel model appears to be a useful framework. It is embedded in sports and considers societal, organizational, and individual factors on the macro, meso, and micro levels for explaining “the attitudes toward and experiences and behaviors of sexual minorities” (Cunningham, 2012, p. 5). The levels are closely interlaced and influence each other in either direction.

On the macro level, cultural norms, and institutionalized practices in society—in which organizations are embedded—influence the situation for transgender persons in sports. The binary gender order and the male hegemony which are reproduced in sports organizations and the rigid sex-segregated competitive structures constitute barriers for transgender participation in organized sports. Transgender athletes are seen “as outsiders or others, because their gender identity did not match the institutionalized ways that sport has been traditionally organized” (Cunningham, 2012, p. 9).

Meso-level factors operate at an organizational level and impact on exclusion and discrimination via leader behavior, organizational culture, and group spirit. The levels of diversity surrounding activities are strongly promoted by the support of organizations' leaders and the allocation of time, resources, and money. Organizational culture reflects a pattern of values, assumptions, and beliefs that have developed over time and are widely shared by the members and serves as an orientation for (in)appropriate behavior (Cunningham, 2012, p. 10). Organizational cultures that assume transgender athletes challenge the superiority of male physicality, distinct gender binaries, and the level playing field impede or complicate inclusion. On the other hand, support within groups on an emotional, structural, and instrumental level fosters the establishment of safe spaces and thus positively impacts on the well-being of transgender athletes (Lucas-Carr and Krane, 2012).

On the micro level, transgender individuals' discrimination in sport can be affected by the personal traits of other participants, bystanders, and spectators. Prejudices against transgender athletes rest upon the widespread assumption of sex binary and heteronormativity and vary between different gender identities, according to how athletes fit into the male-dominated sporting world and the implied (unfair) physical advantages of mainly transgender females (Cunningham and Pickett, 2018; Devís-Devís et al., 2018). Different perceptions of transmen and transwomen in sports are associated with evidence that transgressions of gender norms and gender non-conforming expressions are less socially accepted for and amongst men than women and thus are more likely to provoke discrimination and self-exclusion (Laberge and Albert, 1999). Apart from this, different individual identities, such as gender, racial, cultural, or athletic identity have to be considered when analyzing negative attitudes and behaviors toward transgender individuals. Generally, men articulate more prejudices against sexual minorities than do women in order to ensure male privilege and construct masculinity in heteronormative structures (Cunningham, 2012), although a current study disproves differences in trans prejudices between men and women (Cunningham and Pickett, 2018).

Apart from Cunningham's (2012) multilevel model, Meyer's (2003) minority stress model is valuable for determining the effects of transnegativity. Meyer's model was originally used to explain the high prevalence of mental disorders amongst lesbian women, gay men, and bisexual individuals. Nevertheless, as transgender represent a minority group that is marginalized in dominant cultures and social structures, the model can also be applied for the present target group. In Meyer's (2003) theoretical framework, minority stress is related to environmental circumstances, status and identity of the minority group, different stressors, and social support. Minority stress culminates in stigma, prejudice, and discrimination and can be traced back to distal and proximal stressors. Whilst distal stressors reflect external events and conditions, in which prejudice, discrimination, and violence occur, proximal stressors are subjective “perceptions of the self as a stigmatized and devalued minority” (Meyer, 2003, p. 678) and incorporate expectations of rejection, internalization of transnegativity, and concealment of one's gender identity.

## Transgender Athletes' Experiences

Having laid the theoretical framework for the current study, the quantitative and qualitative research corpus on transgender experiences in sports will be outlined in the following section.

Quantitative research on sports participation, along with the situations and experiences of transgender athletes in different sports and settings, is rare and sometimes inconsistent. Regarding the general sports participation of transgender individuals, Kulick et al. (2018) found fairly equal sports frequencies between transgender and cisgender students in USA, but a mediated, negative effect for sports participation of transgender individuals through feelings of unsafety in facilities (locker-rooms, bathrooms). In contrast, lower participation rates in sports and physical activities were found for transgender people compared to cisgender people in USA (Muchicko et al., 2014) and compared to lesbian women, gay men, and bisexual persons in Canada (ACT Government, 2014).

Personal experiences of transgender athletes in sports, as well as sports-related social and psychological outcomes, are important to determine inclusive or exclusive processes in sports and operate as moderators for (non-)participation (Lucas-Carr and Krane, 2012). In Scotland, eight out of 10 transgender individuals agree that homo-/transnegativity is a major barrier for sports participation and that homo-/transnegativity is a problem in sport (Smith et al., 2012a). Furthermore, there is empirical evidence that most transgender individuals have witnessed or experienced transnegative episodes in sports (Smith et al., 2012a; ACT Government, 2014; Demers, 2017). The numbers are alarming. In Canada, 85% of transgender individuals personally had negative experiences in sports contexts due to their gender identity (Demers, 2017) and in Scotland, 80% of transgender individuals had personally witnessed and/or experienced homo- and transnegativity in sport (Smith et al., 2012a). Referring to all LGBT athletes of the Scottish sample, the share who had personally experienced (17%) or witnessed (49%) homo- or transnegativity in sport is considerably smaller (Smith et al., 2012b). In a large Anglo-American study (USA, Canada, UK, Ireland, Australia, and New Zealand), 82% of the LGB surveyed reported that they have witnessed or experienced homonegative episodes in different sport contexts (Denison and Kitchen, 2015). In the overall OUTSPORT data about 12% of the European LGBTI+ respondents reported personal negative experiences in the past 12 months, with a significantly higher share of non-cisgender people and a slightly increased prevalence in higher performance levels, but independent from the type of sport (individual or team sports) (Hartmann-Tews et al., in press).

The predominant form of experiencing transnegativity is verbal, as almost all transgender individuals experience verbal abuse, insults, disparagement, and offensive remarks in sports (Smith et al., 2012a; Demers, 2017). Referring to homonegativity, Symons (2010) found that verbal abuse toward LGBT athletes most frequently comes from other participants and only a small amount comes from coaches, spectators, or officials. Apart from verbal discrimination, 16% of transgender athletes reported having experienced or witnessed physical abuse and 7% reported other discriminatory experiences related to their gender identity, such as sexual assault (Smith et al., 2012a). After being asked what would help fight discrimination in sport, Scottish transgender respondents mentioned public campaigns, diversity training for different actors in the sport system, and role models (Smith et al., 2012a).

Transgender athletes receive less social support for their physical activities compared to cisgender athletes and share lower scores for several psychological dimensions, such as the perception of their physical self and self-efficacy for exercise behavior (Muchicko et al., 2014). Both social and psychological outcomes are stressors and risk-factors for physical and mental health problems or depression (Symons, 2010; Muchicko et al., 2014).

To draw a comprehensive picture, transgender athletes' narratives about barriers and facilitators for sports participation, as captured in qualitative approaches, will now be discussed.

Mainstream and organized sports activities are mostly experienced as unsafe spaces by transgender individuals, in which they face several distal and proximal stressors (Lucas-Carr and Krane, 2012; Caudwell, 2014; Elling-Machartzki, 2015; Hargie et al., 2017; Jones et al., 2017a; Semerjian, 2019). There is strong academic agreement that the changing room is one of the most challenging barriers: feelings of shame, body incongruence, body dissatisfaction, and fears of others' reactions—all resembling major internal proximal stressors—generate distal stressors, such as abjectification and stigma, negotiations of gender (non-) conformity, and discriminatory behavior (Semerjian and Cohen, 2006; Symons, 2010; Lucas-Carr and Krane, 2012; Smith et al., 2012a; Caudwell, 2014; Elling-Machartzki, 2015; Hargie et al., 2017; Jones et al., 2017a; Kulick et al., 2018; Semerjian, 2019). The locker room is perceived as the most challenging situation, “entrenched in cisgenderism and heteronormativity” (Semerjian, 2019, p. 154), but the entanglement of proximal and distal stressors generally leads to feelings of fear in public sporting spaces, which often impede sports engagement (Hargie et al., 2017). Moreover, alienating negative experiences concerning physical education in school are major barriers for further sports participation of transgender individuals (Caudwell, 2014; Hargie et al., 2017). Also, clothing regulations and norms, participation in sex-segregated team sports, and a lack of acceptance in teams pose further external barriers to transgender sports participation (Jones et al., 2017a; Semerjian, 2019).

Further exclusionary processes toward transgender athletes, discovered by Hargie et al. (2017), provide supporting evidence for Bailey's (2005) dimensions of social exclusion. In regard to organizational sports settings, Hargie et al. (2017, p. 234) found that hostility from facility staff and members of the public emerged as relational stressors; high social, and economic distances led to a lack of social networks; social capital acted as spatial stressors; missed opportunities for gaining knowledge, competencies, and capabilities, as well as refraining from specific sports activities, occurred as functional stressors; and internalized transnegative attitudes and reduced self-confidence arose on the power dimension.

Despite the barriers outlined, transgender perspectives stress the positive consequences of sports participation. Being physically active and doing sports contribute to body awareness and satisfaction, gender (dis-)identification, and recognition and accentuation of body changes (Elling-Machartzki, 2015; Jones et al., 2017a; Semerjian, 2019). Positive effects from sports on mental well-being and social involvement in welcoming, safe, and comfortable sports settings strengthen transgender individuals' abilities to cope with challenges (Elling-Machartzki, 2015; Elling and Collot d'Escury, 2017). Several studies have identified safe spaces in sport, in which transgender athletes were accepted, socially supported by the team, and enabled to compete without rigid restrictions (Lucas-Carr and Krane, 2012, Travers and Deri, 2010; Elling and Collot d'Escury, 2017). But as these conditions are rather rare in mainstream sports settings, establishing trans-only sporting environments might externally facilitate transgender sports participation (Jones et al., 2017a).

Reflecting on the findings regarding barriers and facilitators for transgender sports participation, it should be mentioned that experiences are diverse, individual, and often dependent on settings, sports, social environment, or personal traits (Semerjian and Cohen, 2006). Nevertheless, most transgender athletes are confronted with prejudices and ignorance in sports and personally experience discrimination, harassment, and scarce access to safe, comfortable, and inclusive sports environments (Jones et al., 2017b). Exclusionary mechanisms are based on the supposed incongruence of transgender individuals with the dominant assumption of sex binary and the sex-segregation in organized sports settings (Symons, 2010; Lucas-Carr and Krane, 2012; Elling and Collot d'Escury, 2017) and culminate in the “overall effects of being denied the social, health and well-being aspects of sport” (Hargie et al., 2017, p. 223). Elling-Machartzki (2015) emphasizes that traditional sports clubs appear to be particularly challenging sports settings due to the changing room situation and discriminatory policies for engaging in the competitive system.

## Organizational Policies on Transgender Athletes' Inclusion

(Sport) clubs are prototypes of self-purpose organizations with the main function to serve the interests of their members and trigger them to engage in specific (sport) activities (Müller-Jentsch, 2008). Apart from that, in the sense of Ferdinand Tönnies sport clubs can promote communitization in society as well as social change (Müller-Jentsch, 2008) and are perceived as drivers for social inclusion. This has become evident in manifold sport for all campaigns or recent diversity management approaches in organized sports (Rulofs, 2012). However, as sports clubs have a considerably high “resistance to change” (Cunningham, 2007, p. 306), inclusion of underrepresented groups is a tremendous challenge. Especially if these groups challenge the logic inherent in the sport system, such as sex binary or the level playing field. Therefore, barriers for transgender sports participation are not only situated on personal and interactional levels but on institutional and policy levels (Symons, 2010; Caudwell, 2014; Jones et al., 2017b). In their systematic review of transgender persons' experiences of sports and organizational policies regarding transgender inclusion, Jones et al. (2017b, p. 712) emphasize that “the requirements that transgender competitive sport policies place on competitors were instrumental in transgender athletes' negative experiences,” even in recreational sports settings.

To deal with transgender athletes in competitive sports, Griffin (2012, p. 107f) outlined four potential policy forms: (1) use of mandatory sex-verification testing to maintain the gender binary; (2) case-by-case decisions on transgender inclusion using sex-verification testing; (3) implementation of categories other than sex [i.e., Kane's (1995) idea of a “continuum, whereby sport is organized based on ability and athletes compete in skill level groupings, regardless of gender” (cited after Lucas-Carr and Krane, 2012, p. 39)]; and (4) expansion of gender categories to include transgender athletes. Many current sports organizations' policies belong to Griffin's (2012) second category and are perceived as discriminatory for transgender athletes; more inclusive policies are mainly represented in the fourth category.

The International Olympic Committee (IOC) policy 2004, known as the Stockholm Consensus, was one of the first attempts to regulate transgender participation and has been fully or partly (for transgender females) adopted by sports organizations worldwide (e.g., British Swimming Association, 2015; USA Gymnastics, 2015; Fédération Internationale de Volleyball, 2019). This policy requires of athletes a gender-confirming surgery, hormone treatment, and living in the gender identity, each for at least 2 years (International Olympic Committee, 2004). Since hormones are supposed to have strong effects on physicality, some organizations' policies require different degrees of hormone treatment and different threshold levels besides the legal recognition of gender identity (e.g., The Football Association, 2014; International Association of Athletics Federations, 2018, 2019; International Tennis Association, 2018; International Cycling Union, 2020). Criticism of these rigid transgender policies centers on the exclusion of transgender athletes who are not undergoing medical treatment; who are just in the process of transitioning; whose gender identity lies outside the gender binary; and on the discrimination toward transgender males who have allegedly no physical advantages (Lucas-Carr and Krane, 2011; Jones et al., 2017b; Semerjian, 2019).

Taking some of the reservations and criticisms into account, in 2015 the IOC implemented different policies for male and female transgender individuals. For female transgender athletes, the requirement demands 4 years of declared gender identity and a threshold for testosterone levels, whereas male transgender athletes can compete without any restrictions (International Olympic Committee, 2015; e.g., USA Swimming, 2018; Badminton England, 2019). The National College Athletics Association has already implemented such policies in 2011 and, beyond that, allows transgender athletes to stay on their teams, as long as they do not start medical, hormonal transformation (National College Athletics Association, 2011). These policies were also implemented by the National Women's Hockey League (2016) for elite athletes. More inclusive policies only require legal recognition of gender identity (US Soccer Federation, 2013; Berliner Fußball Verband, 2018; USA Sailing, 2019) or allow participation simply on the basis of gender identity, regardless of legal recognition, medical treatment, and anatomy (UK Roller Derby Association, 2014; International Quidditch Association, 2018; Washington Interscholastic Activities Association, 2019).

Travers and Deri (2010, p. 503) articulate two barriers for inclusive transgender policies: first, the “powerful culture of sex binary logic in the organization of sport,” built on the assumed relevance of testosterone; and second, obstacles to “re-negotiate sexed boundaries,” related to the dissent amongst transgender athletes about appropriate inclusive policies. Jones et al. (2017b) question the inclusiveness of the reviewed policies, concluding that the majority of the applied policies in different organized sports institutions discriminate against transgender individuals and most notably transgender males, mainly because no empirical evidence exists on the athletic advantages of female and male transgender athletes. This is empirically supported by Harper (2015), who identified times for middle distance runs for transgender females that comparably change according to the bodily status of adaption. From a philosophical point of view, Gleaves and Lehrbach (2016) call for society to focus on “narrativity rather than physiological equivalency,” so as to include transgender athletes, instead of perceiving sport solely as a comparison of physical skills and competencies.

## Methodology

As the theoretical framework and research corpus have shown, transgender athletes are a highly controversial topic, encompassing inherent sports values such as (un)fairness, in-/exclusion, (non)discrimination, and (un)equal opportunities. Thus, inclusion of transgender athletes in organized sports often fails, which leads to discriminatory experiences, humiliation, exclusion, and stress. This paper has three objectives: (1) to analyze the LGBT+ athletes' assessment of transnegativity in organized sports settings in Europe; (2) to scrutinize negative experiences (prevalence, forms, perpetrators) with regard to gender identity, performance level, and LGBT+ reference in organized sports settings in Europe; and (3) to discuss inclusive strategies, as well as barriers and facilitators, for implementing diversity in sports organizations in Europe. The first and the second objective are explored through a quantitative online survey in all European Union countries, and the third objective is explored through a qualitative interview study in five European countries (GER, ITA, AUT, SCO, HUN). Both studies were conducted as part of the European joint research project OUTSPORT, which was developed by the German Sport University Cologne in cooperation with the Italian Association for Culture and Sport (AICS), LEAP Sports Scotland (LEAP), the Vienna Institute for International Dialogue and Cooperation (VIDC), and the Organization for Fresh Ideas, Hungary (FRIGO). In contrast to further publications already issued within the framework of this project (e.g., Menzel et al., 2019; Hartmann-Tews et al., in press), the current paper focuses on: (1) subsample of LGBT+ athletes within organized sports contexts, (2) systematic differentiation between cisgender and non-cisgender athletes, and (3) integration of qualitative interviews with representatives of sports organizations.

### Athletes' Experiences: Quantitative Online Survey

#### Sample

The target population of the OUTSPORT survey was defined as anyone who (a) identifies as lesbian, gay, bisexual, transgender, and/or intersex; (b) currently lives in one of the 28 member states of the European Union; and (c) is at least 16 years old. Being currently active in sports was not set as a mandatory criterion. Due to stigmatization, discrimination, and the lack of knowledge about socio-structural parameters—particularly on a European level—LGBT+ populations can be characterized as hard-to-reach, hidden, and vulnerable populations (Ellard-Gray et al., 2015). In order to draw a systematic sample of this population, a combination of sampling strategies was applied. Primarily, various LGBT+ organizations in each of the 28 EU member states were contacted and asked for contact details from other LGBT+ (sports) organizations and umbrella sports organizations in their countries. Each of these organizations was asked for support and supplied with standardized packages of promotional material (texts, pictures, videos) to advertise the web-based survey via their channels (i.e., social media, web pages, mailing lists). International LGBT+ organizations such as the European Gay and Lesbian Sport Federation and the International Lesbian, Gay, Bisexual, Trans and Intersex Association also helped to promote the survey. To increase the reach, a snowball recruiting technique among participants was applied. Social media targeting was used in an adaptive process to balance out lower participation rates in specific countries. The survey was promoted in a neutral way. To avoid a negativity bias, words and expressions such as “negative experiences,” “discrimination,” or “harassment” were not used. The online survey was accessible in German, English, Italian, Hungarian, and French. All translations were conducted by ETC Europe, a professional translation agency. To improve validity and reliability, emphasis was placed on precise wording in each language. Sensitive wordings were thoroughly double-checked by native speakers with an LGBT+ background who also worked for the OUTSPORT project. Anonymity and confidentiality for the participants were secured, and the General Data Protection Regulation of the EU was applied. The research design was approved by the ethics committee of the German Sport University Cologne. The online survey was accessible between March and August 2018.

The total sample contains 5,524 valid cases. The share of each country's respondents from the total sample roughly corresponds to the share of each country's inhabitants from the total EU population (mean deviation of percentage points: M = 1.68, SD = 0.019). Within the scope of this paper, the focus is placed on a subsample of active respondents who practice their (main) sport in an organized sports context (*n* = 2,282). This could either be organized sports clubs (*n* = 1,391), for-profit organizations such as fitness centers (*n* = 624), or other organizations (e.g., company or university sporting groups etc.) (*n* = 267). Participants from the chosen subsample are between 16 and 75 years old (M = 27.3, SD = 11.2). More than half (51%) have a college or university degree, and more than a third (37%) have completed upper or post-secondary education. Almost two thirds live in urban areas with over 100 k inhabitants (64%), one out of five (19%) lives in towns or smaller villages (20–100 k), and 17% live in rural areas (<20 k). The assigned sex at birth was female in 60% of cases and male in 40% of cases.

#### Measures

Based on the relevant research corpus the questionnaire for the quantitative online survey has been developed by the authors in cooperation with experts from the OUTSPORT project team. To ensure validity and rigor, the questions have been further discussed with scientific experts inside and outside academia and a pre-test has been performed leading to further adjustments. The following section shows precisely how the variables used in this paper have been measured and operationalized.

##### Gender identity

A two-question gender status measure was used to retrieve data about the gender identity of respondents (Tate et al., 2013; Lombardi and Banik, 2016). In a first step, respondents were asked to report their sex assigned at birth, with the options^3^ (1)“male” and (2) “female,” before respondents were asked about their current gender identity: (1)“female”; (2)“male”; (3)“transgender”; and (4)“I do not identify myself as male, female or transgender.” Cross-analysis of these two questions resulted in six distinct gender-identity categories: female cisgender, male cisgender, female transgender, male transgender, non-binary transgender, and non-identifying people (respondents who do not identify as male, female, or transgender). Within the scope of this paper, the main distinction made will be between cisgender and non-cisgender respondents. Non-cisgender respondents will be further subdivided into three groups: female transgender; male transgender; and non-binary respondents (including non-identifying respondents).

##### Sports setting

In addition to the organizational context of a sport (e.g., sports clubs, for-profit organizations, or other organizations), two more dimensions are used to describe the sports setting: performance level and LGBT+ reference. Performance level was assessed by a single choice question (“What was the nature of this activity?”), offering the response options (1)“recreational”; (2)“competitive”; and (3)“high performance.” The LGBT+ reference of a sports activity was assessed by asking respondents to characterize their sports context as either (1)“mainstream/not specified”; (2)“explicitly LGBT+ friendly”; or (3)“LGBT+ specific.”

##### General assessment of transnegativity in sports

To assess the perception of transnegativity in sports on a general level, respondents were asked if they think that there is a problem with transnegativity in sports. Response options were provided on a five-point scale from 1 (“no problem”) to 5 (“big problem”) and a no-choice option (“don't know”). Transnegativity was defined in the questionnaire as “prejudice or discrimination on grounds of transgender identity.”

##### Helpful strategies and awareness of contact points

Respondents were asked what they would consider appropriate measures to tackle discrimination or harassment in sports due to sexual orientation and/or gender identity. To find out how aware people are of whom to turn to in cases of homo-/transnegative incidents in their sport, respondents were asked whether they know any support contact points (organizations or individuals) they can get in touch with if such an incident happens.

##### Homo-/Transnegative incidents

Homo-/transnegative incidents were assessed in a two-step approach. First, respondents were asked whether or not (“yes”/“no”) they had personal negative experiences in their sport during the previous 12 months: “Looking back at the last 12 months, did you personally have any negative experiences in this specific sports context as a result of your sexual orientation and/or gender identity?” The question was formulated such that the connection to a specific sports activity and its context, the causal attribution to the respondent's own sexual orientation and/or gender identity, and the topicality (last 12 months) were emphasized. In the second step, respondents were asked how often they had personally experienced several forms of homo-/transnegative incidents in that time span: (1) verbal insults and slurs (ridiculing, name-calling, derogatory words such as “dyke,” “faggot,” “poofter” etc.); (2) verbal threats (involving harm and/or intimidation); (3) physical crossing of lines (shoving, pushing, inappropriate touching etc.); (4) physical violence (kicking, punching, deliberate injuring; sexual assaults etc.); (5) discrimination (unfair treatment, exclusion, unequal opportunities etc.); (6) e-bullying (harassment via social media, messengers, webpages); and (7) other (“please specify”). Respondents were provided with a five-point scale response option (1 = “never,” 5 = “very often”) for each form. Besides the forms and frequencies, respondents were asked about the perpetrators. For every form of homo-/transnegative incidents that occurred, respondents were asked, “Who said or did this?,” offering seven different perpetrators via a multiple-choice option.

### Organizational Strategies: Qualitative Interview Study

The qualitative study consists of 15 expert interviews with relevant stakeholders from sport systems in the OUTSPORT project partner countries Germany, Scotland, Italy, Austria, and Hungary. The interviewees represent responsible actors from umbrella sports organizations of the voluntary sector at national or regional levels (DOSB, LSB, CL, SS, UISP, AICS, BSO, AGC, MOB, MA), specific sports federations (bfv, “FA,” FIR), and public sports organizations focusing on specific target groups (SDS, SSS) (Table 1). The semi-structured, guided interviews focused on the broader context of LGBT+ inclusion in sport and organizational strategies for diversity and inclusion. In order to identify facilitators for implementing diversity strategies and inclusive policies for LGBT+ minorities in sport, the sampling aimed at including good practice organizations.

**Table 1:** Participating sports organizations from project partner countries (Germany, Scotland, Italy, Hungary, Austria).

Germany	German Olympic Sports Confederation	DOSB	Italy	Italian Sport Union for all	UISP
	State Sports Confederation of Saxony-Anhalt	LSB		Italian Culture Sport Agency	AICS
	Football Association Baden	Bfv		Italian Rugby Federation	FIR
	Football Association (anonymous)	FA			
Scotland	Community Leisure UK—Scotland	CL	Austria	Austrian Sport Organization	BSO
	Sportscotland	SS		Austrian Center for Gender Competence	AGC
	Scottish Disability Sport	SDS	Hungary	Hungarian Leisure Sport Association	MOB
	Scottish Student Sport	SSS		Hungarian Olympic Committee	MA

The qualitative data collection was carried out between September 2018 and April 2019. The Scottish, Italian, and Hungarian interviews (along with one Austrian interview) were conducted and transcribed by the respective project partner; one Austrian interview and the German interviews were conducted by the authors. All interviews were coded by the authors using qualitative, category-led text analysis (Mayring and Fenzl, 2014). Deductively and inductively developed (sub-)categories structured the coding process and the interpretation of the findings; both approaches were critically discussed with colleagues to ensure trustworthiness of the data. For the current purpose, findings on barriers and facilitators for implementing inclusive diversity strategies in sports organizations and the parts on transgender issues are focused on. Due to methodological issues in conducting interviews and different stages concerning problem awareness and inclusiveness for LGBT+ athletes in the five project partner countries, the results on transgender issues focus mainly on interviews from Germany and Scotland.

## Empirical Results

Before the results are going to be presented in detail, the sports participation of the respondents will be briefly described.

For findings on the general sports participation of different gender-identity groups, the overall OUTSPORT sample (*n* = 5,524) was used. It was found that cisgender and non-cisgender respondents differ slightly in their general sports participation over the last 12 months. Namely, transgender males (68%) have the highest share of active respondents, followed by both cisgender males and females (63%), followed by female and non-binary non-cisgender respondents (57/59%) (Menzel et al., 2019).

In the current sample of active respondents in organized sport (*n* = 2,282), six out of 10 respondents (61.0%) practice their main sport activity in organized sports clubs, 27.3% practice in for-profit organizations, and 11.7% practice in other organizations such as university or company sporting groups. Cisgender individuals participate more often in organized sports clubs, while non-cisgender individuals engage more often in activities held by other organizations (χ^2^(2) = 12.99, *p* < 0.01, V = 0.075). The performance level is predominantly recreational (56.5%), while competitive (31.6%) and high-performance (12.0%) levels are scarcer. Eight out of 10 (82.6%) respondents practice their sport in mainstream or LGBT+ unrelated settings and 17.4% practice in LGBT+ related settings—either explicitly LGBT+ friendly (9.1%) or LGBT+ specific (8.3%). With regard to the performance level, no significant differences were found between cisgender and non-cisgender respondents (χ^2^(2) = 2.67, n.s.). The share of cisgender respondents in LGBT+ specific settings (9.0%) is slightly higher compared to non-cisgender respondents (3.9%) (χ^2^(2) = 10.85, *p* < 0.01, V = 0.069).

### Transnegativity in Sport

Transnegativity is perceived to be a “big problem” by the majority of respondents. On the 5-point scale (1 = “no problem,” 5 = “big problem”), 88.9% of respondents chose either category four (25.9%) or five (63.0%), causing a strongly left-skewed distribution^4^. Cisgender (M = 4.47, SD = 0.81) and non-cisgender (M = 4.50, SD = 0.87) respondents show similar mean values, with no significant differences in mean ranks (U = 273199.5, n.s.). Within the group of non-cisgender athletes, respondents with a non-binary gender identity perceive transnegativity to be a bigger problem (M = 4.58, SD = 0.80) than do female (M = 4.17, SD = 1.29) and male (M = 4.24, SD = 0.95) transgender athletes. Accordingly, a Kruskal-Wallis test shows significant differences of means (H(2) = 9.47, *p* < 0.01), with mean rank scores of 144.11 for transgender females (*n* = 18), 133.3 for transgender males (*n* = 54) and 166.99 for non-binary persons (*n* = 247). *Post-hoc* tests of pairwise comparisons show significant group differences between male transgender and non-binary respondents (*p* < 0.01; r = 0.169), with a medium effect size.

### Personal Negative Experiences

Of all active LGBT+ respondents in organized sports settings, 11.8% have had at least one negative personal experience that was associated with their sexual orientation or gender identity in their main sport in the last 12 months (Table 2). The differences between cisgender and non-cisgender respondents are remarkable. The share of non-cisgender athletes who have become victims of homo-/transnegative incidents is three times higher (29.6%) compared to cisgender athletes (8.8%) (χ^2^(2) = 118.22, *p* < 0.001, V = 0.228). This effect consistently occurs in all three performance levels and all LGBT+ reference types with weak to moderate effect sizes (Table 2). Irrespective of respondents' gender identity, higher rates of homo-/transnegative incidents are weakly associated with higher performance levels (χ^2^(2) = 9.66, *p* < 0.01, V = 0.068) and more likely to occur in mainstream than in LGBT+ related settings (χ^2^(2) = 6.67, *p* < 0.05, V = 0.053). Among non-cisgender respondents, differences with regard to the performance level (χ^2^(2) = 4.01, n.s.) and LGBT+ reference (χ^2^(2) = 1.64, n.s.) are not statistically significant.

**Table 2:** Personal negative experiences due to SOGI in different settings by gender identity (%).

	**Total**	**CIS**	**Non-CIS**	**χ^2^**_**(1) columns**_ _**(*asymptotic*)**_	**Cramer's V**	***n***
**Total**	11.8	8.8	29.6	118.22^***^	*0.228*	*2,282*
**Performance level (*****n*****)**						
Recreational	10.3	7.4	26.6	67.39^***^	*0.229*	*1,286*
Competitive	12.5	9.9	30.4	30.93^***^	*0.207*	*719*
High performance	16.8	12.4	42.5	22.01^***^	*0.284*	*273*
*χ^2^_(2)*rows* (*exact*)_*	9.66^**^	7.58^*^	*4.01*			
*Cramer's V*	*0.068*	*0.063*	–			
*n*	*2,278*	*1,947*	*331*			
**LGBT+ reference (*****n*****)**						
Mainstream	12.4	*9.4*	30.0	93.12^***^	*0.223*	*1,881*
LGBT+ friendly	10.6	*8.2*	21.6	5.73^*^	*0.166*	*207*
LGBT+ specific	6.4	*4.0*	38.5	24.05^***^	*0.358*	*188*
*χ^2^_(2)*rows* (*exact*)_*	6.67^*^	6.20^*^	*1.64*			
*Cramer's V*	*0.053*	*0.054*	–			
*n*	*2,276*	*1,946*	*330*			

*Question: “Looking back at the last 12 months, did you personally have any negative experiences in this specific sports context as a result of your sexual orientation or gender identity?” Database: Active sports participants in organized sports*.

*Test statistics (χ^2^) and effect sizes (Cramer V) are written in italic*.

* *p ≤ 0.05*;

** *p ≤ 0.01*;

*** *p ≤ 0.001*.

Among those who have personally experienced homo-/transnegative incidents in the last 12 months, verbal insults (79.2%) and structural discrimination such as unequal opportunities, unfair treatment, or exclusion (75.1%) were the most common forms (Table 3). Moreover, verbal threats and intimidations occurred in 39.4% of the cases, and harassment via social media, messengers, or webpages (e-bullying) occurred in 35.3% of cases. Physical types of homo-/transnegative incidents happened to 31.6% of respondents as lighter forms of crossing the lines (e.g., shoving, pushing, or inappropriate touching) and to 15.6% as severe forms of physical violence (e.g., kicking, punching, or injuring). For reasons of clarity, the 5-point scaled data was dichotomized first. Percentages are used to indicate the share of respondents who experienced specific types of incidents.

**Table 3:** Forms of personal negative experiences by gender identity, performance level, and LGBT+ reference (%).

		**Gender identity**			**Performance level**				**LGBT+ reference**		
	**Total**	**CIS**	**Non-CIS**	**χ^**2**^_**(1)**_**	**R**	**C**	**HP**	**χ^**2**^_**(2)**_**	**Main-stream**	**LGBT+**	**χ^**2**^_**(1)**_**
Verbal insults	79.2	86.0	67.3	13.10^***^	72.9	83.3	89.1	6.85^*^	79.1	82.4	*0.20*
Discrimination	75.1	71.3	81.6	3.53^*^	71.4	77.8	80.4	*2.00*	76.1	67.6	*1.12*
Verbal threats	39.4	39.2	39.8	*0.01*	37.6	42.2	39.1	*0.48*	39.7	38.2	*0.03*
E-bullying	35.3	37.4	31.6	*0.92*	36.1	31.1	41.3	*1.45*	36.3	29.4	*0.62*
Physical crossing of lines	31.6	33.3	28.6	*0.65*	27.1	37.8	32.6	*2.88*	32.1	29.4	*0.10*
Physical violence	15.6	14.6	17.3	*0.35*	15.0	18.9	10.9	*1.55*	14.1	26.5	*3.44*
Other	11.5	7.6	18.4	7.08^**^	19.5	4.4	2.2	16.77^***^	10.7	17.6	*0.20*
*N*	269	171	98		133	90	46		234	55	

*Question: “In the last 12 months, how often did you personally experience the following as a result of your sexual orientation or gender identity?” Database: Active sports participants in organized sports who have had personal negative experiences associated with their sexual orientation or gender identity in the last 12 months*.

*Test statistics (χ^2^) and effect sizes (Cramer V) are written in italic*.

* *p ≤ 0.05*;

** *p ≤ 0.01*;

*** *p ≤ 0.001*.

Table 3 shows group comparisons regarding gender identity, performance levels, and LGBT+ reference of the sport. Results indicate that verbal insults are more frequently experienced among cisgender respondents (χ^2^(2) = 13.10, *p* < 0.001, V = 0.221) and in higher performance levels (χ^2^(2) = 6.85, *p* < 0.05, V = 0.160). Non-cisgender respondents more commonly experience structural discrimination (χ^2^(2) = 3.53, *p* < 0.05, V = 0.114) and other negative incidents (χ^2^(2) = 7.08, *p* < 0.01, V = 0.162), which are typically associated with misgendering (i.e., problems concerning the use of correct pronouns and appropriate naming) or being looked at in a derogatory way. No statistically significant differences were found between mainstream and LGBT+ related sport settings, although a higher rate of physical violence is observed in LGBT+ related settings (χ^2^(1) = 3.44, *p* = 0.064; V = 0.113).

For each of the seven forms of homo-/transnegative incidents, respondents were asked about the perpetrators (“Who said or did this?”), with multiple-choice options. Table 4 shows the percentages for all seven forms combined (i.e., the share of respondents who named the corresponding perpetrator at least once in any of the occurring forms). Members of respondents' own teams (54.9%) and other sport participants (51.4%) are the most frequently mentioned perpetrators, irrespective of the specific types of incidents. About a third of respondents with homo-/transnegative experiences identified members of opposition teams (36.1%), coaches (34.1%), and spectators (32.5%) as perpetrators, whereas about one out of six indicated that at least one negative incident was caused by other officials (17.3%).

**Table 4:** Perpetrators by gender identity, performance level and LGBT+ reference (%).

		**Gender identity**			**Performance level**				**LGBT+ reference**		
	**Total**	**CIS**	**Non-CIS**	**χ^**2**^_**(1)**_**	**R**	**C**	**HP**	**χ^**2**^_**(2)**_**	**Main-stream**	**LGBT+**	**χ^**2**^_**(1)**_**
Team members	54.9	53.0	58.2	*0.64*	42.1	64.7	72.7	17.33^***^	57.9	36,4	5.39^*^
Other participants	51.4	51.8	50.5	*0.04*	63.5	42.4	34.1	15.44^***^	52.0	45,5	*0.50*
Op. team members	36.1	37.8	33.0	*0.59*	25.4	52.9	34.1	16.79^***^	34.8	45,5	*1.40*
Coaches	34.1	28.7	44.0	6.09^*^	36.5	30.6	34.1	*0.79*	34.8	30,3	*0.26*
Spectators	32.5	31.7	34.1	*0.15*	33.3	34.1	27.3	*0.69*	30.3	48,5	4.31^*^
Other officials	17.3	14.6	22.0	*2.21*	21.4	12.9	13.6	*3.05*	15.4	30,3	4.46^*^
Other	6.3	5.5	7.7	*0.48*	7.9	3.5	6.8	*1.70*	6.3	6,1	*0.00*
*N*	255	164	91		126	85	44		221	33	

*Question: “Who said or did this?” (Combined for all seven forms of homo-/transnegative incidents). Database: Active sports participants in organized sports who have had personal negative experiences associated with their sexual orientation or gender identity in the last 12 months and who named at least one perpetrator (n = 255)*.

*Test statistics (χ^2^) and effect sizes (Cramer V) are written in italic*.

* *p ≤ 0.05*;

** *p ≤ 0.01*;

*** *p ≤ 0.001*.

Cisgender and non-cisgender respondents referred to roughly the same types of perpetrators except for coaches, who are more commonly indicated by non-cisgender respondents (χ^2^(2) = 6.09, *p* < 0.05, V = 0.115). Highly significant differences can be found with regard to the performance levels. Perpetrators from respondents' own teams (χ^2^(2) = 17.33, *p* < 0.001, V = 0.261) or opponent teams (χ^2^(2) = 16.79, *p* < 0.001, V = 0.257) were more frequently indicated by athletes in higher performance and competitive levels, respectively. In contrast, other sports participants were more frequently referred to as perpetrators in recreational settings (χ^2^(2) = 15.44, *p* < 0.001, V = 0.246). Spectators (χ^2^(1) = 4.31, *p* < 0.051, V = 0.130) and other officials (χ^2^(1) = 4.46, *p* < 0.05, V = 0.133) were mentioned significantly more often by respondents in LGBT+ related sports settings, whereas team members as perpetrators are more widespread in mainstream sports settings (χ^2^(1) = 5.39, *p* < 0.05, V = 0.146).

### Tackling of Transnegativity

Respondents who have had homo-/transnegative experiences at some point in their sport (*n* = 616) were asked whether they were aware of contact points for support (i.e., organizations and/or individuals concerned with matters of discrimination). One out of three respondents (32.3%) indicated being aware of contact points in non-governmental organizations outside of the organized sport. One out of five respondents (19.2%) was aware of contact points in local sports organizations, and one out of seven was aware of contact points in umbrella sports organizations on a regional or national level (14.4%). There are no statistically significant differences between cisgender and transgender respondents.

The most frequently named measures to tackle discrimination and harassment on the grounds of sexual orientation and gender identity are to encourage more sports stars to come out and to run high-profile anti-homophobia/transnegativity campaigns, followed by diversity training and more inclusive policies (Table 5)^5^. More than two thirds of respondents considered tougher sanctions as appropriate measures, whereas almost 9% referred to other measures. The coming-out of sport stars is perceived to be helpful by a higher share of cisgender respondents (χ^2^(1) = 7.32, *p* < 0.01, V = 0.100). Non-cisgender respondents consider inclusive policies (χ^2^(1) = 22.35, *p* < 0.001, V = 0.175) and diversity training (χ^2^(1) = 5.67, *p* < 0.05, V = 0.088) more important and also refer to other strategies twice as often as cisgender respondents (χ^2^(1) = 9.76, *p* < 0.01, V = 0.115). Better education about gender diversity, gender-neutral changing rooms, and enhancement of social acceptance were most frequently mentioned.

**Table 5:** Helpful to tackle discrimination in sport by gender identity.

	**Total**	**CIS**	**Non-CIS**	**χ^**2**^_**(1)**_**	**Cramer's V**
Sports stars coming out	70.1	72.5	61.3	7.32^**^	*0.100*
Anti- H/T campaigns	67.7	67.8	67.1	*0.03*	*-*
Diversity training	62.8	60.6	71.0	5.67^*^	*0.088*
Inclusive policies	50.9	46.4	67.7	22.35^***^	*0.175*
Tougher sanctions	35.9	35.6	36.8	*0.07*	*-*
Other	8.6	6.9	14.8	9.76^**^	*0.115*
N	733	578	155		

*Question: “What do you think would be helpful in tackling discrimination and/or harassment based on sexual orientation and/or gender identity in sport?” Multiple choice, figures in valid percentages. Database: Active sports participants in organized sports*.

*Test statistics (χ^2^) and effect sizes (Cramer V) are written in italic*.

* *p ≤ 0.05*;

** *p ≤ 0.01*;

*** *p ≤ 0.001*.

### Transgender Issues in Sports Organizations

The issue of transgender inclusion in sports is an ongoing and complex topic for organizations in Germany and Scotland, while in the other countries, the organizational approach to the issue of transgender athletes appears relatively vague and sometimes unpopular. In relation to the engagement of several sports federations with transgender issues, the interviewee from the Austrian Center for Gender Competence pointed out that “it's rather an unpopular topic that they have to deal with, not want to deal with” (AGC, 51). Thus, the German football associations interviewed, as well as organizations in Scotland, reported that transgender inclusion into competitive systems is mainly ruled by case-by-case decisions and individually by the responsible organization. Some positive examples of regulations were mentioned in the interviews, as the following quote from sportscotland indicates:

Gymnastics made a regulation change because the rules stated that females had to wear leotards, a certain outfit, and there was a young transwoman who didn't feel comfortable with that. And she actually was getting deducted points because she wanted to wear different clothing. Now again that's something very simple but was creating a massive issue for her competition (SS, 419–422).

The German and Scottish organizations highlight the need for implementing general inclusive policies on a broader level, and in most of the organizations, there are already efforts to develop generally binding regulations and guidelines. The ruling by the Federal Constitutional Court in Germany on the option of a third gender (divers) has increased the urgency for inclusive policies beyond the sex binary, as the German Olympic Sport Federation emphasized: “especially in the context of grassroots sport, national organizations are invited to formulate appropriate recommendations” (DOSB, 481–482).

The organizations in Germany and Scotland perceive both cooperation within the sport system and low-threshold offers as beneficial. Workshops, events, and other opportunities enable different actors in the sport system to get in touch with each other and discuss various ways for transgender inclusion into competitive sport structures. Mutual learning and inspiring each other is beneficial, as the Football Association Baden stressed: “the topic is not about competition or who does what better or something like that, but it's simply about mutual stimulation (…) and motivating people to move forward with the topic” (bfv, 155–157).

### Facilitators for Implementing Diversity Strategies

Apart from specific issues regarding transgender athletes, the 15 interviews focused on the general inclusion of LGBT+ athletes in sport organizations. With this broader focus, the barriers and facilitators for implementing diversity strategies will be discussed with reference to Cunningham's (2012) multilevel model.

On the macro level, the majority of sporting organizations demand that politicians and the public lead the way to LGBT+ inclusion, address the situation, and raise awareness about the barriers for the inclusion of LGBT+. These external factors were perceived as beneficial for implementing the topic of diversity in sports organizations. Many interviewees perceived hardly any rejection of the topic but rather perceived a lack of knowledge and awareness, as highlighted by sportscotland: “If you're looking at resistance, the only thing we tend to come across is lack of awareness and education” (SS, 216–217). The Italian Culture Sport Agency perceived some areas of resentment, but only when this agency started to deal with LGBT+ issues: “And even those cultural resistances that existed at the beginning (…), slowly absorbed this new situation and have accepted it, and today frankly LGBT issue is not a sector we put a “different” effort in, it is a sector like all the others” (AICS, 25–26). In contrast, however, the Hungarian Olympic Committee expected rejection of their member organizations when addressing the issues of LGBT+ inclusion: “But our member clubs are independent; we have no legal ways to interfere. What we can change is the mentality. But if we start to deal with these issues, there will be problems” (MA, 20–22). Therefore, communication, education, and role models serve as important tools for raising awareness and increasing sensitivity to the issue of diversity and inclusion of sexual minorities in sports. The interviewee from the Italian Sport Union For All emphasized this: “The fact that there are athletes who come out is very important (…) as well as the fact that there are more and more moments where various subjects and institutions address LGBT issues and try to question themselves on this matter” (UISP, 38–39).

Moreover, male hegemony that is reproduced on various levels in sports is perceived as a barrier for LGBT+ inclusion. Individuals who are not representing heteronormative and male stereotypes do not fit into sporting realities as the interviewee from the Austrian Center for Gender Competence stressed: “I think anything that runs counter to the typical male ideal is simply insecure in sport. Women, lesbians, gays, transgender people, girls in headscarves, people with disabilities” (AGC, 368–371).

On the meso level, cooperation with governing bodies, along with relevant stakeholders inside and outside the sport systems, served as an important facilitator for the integration of LGBT+ issues into sports clubs and organizations. Furthermore, a welcoming organizational culture and robust inclusive values were mentioned as instrumental for the inclusion of LGBT+ athletes: “We definitely live and breathe our values (…) we try and theme things around them. So, our values are at the core of everything we do” (Scottish Student Sport, 266–269).

The organizations believe that both top-down and bottom-up strategies are needed to implement LGBT+ inclusion. The interviewee from the State Sports Confederation of Saxony-Anhalt considers umbrella organizations as important role models for implementing the topic: “It is also important, of course, that the umbrella organizations take the appropriate steps, because then, I believe, it will be easier for the smaller associations, organizations, and clubs to support the issues, if the umbrella organizations put them into practice” (LSB, 919–922). However, the implementation of LGBT+ inclusion solely via top-down strategies in sport might ignore “barriers of volunteering, lack of time, and lack of personnel” (BSO, 313–314) that most sports clubs experience, as the Austrian Sport Organization mentioned.

The organizations emphasized the need for a distinct commitment of the organizations' leadership to the topic of diversity and inclusion, along with the willingness to act and to allocate respective resources for initiating activities and implementing strategies. Conversely, a lack of commitment and a lack of financial, staffing, and time resources are viewed as major barriers for implementing strategies, campaigns, or other measures. “I suppose the only barrier that we have is the size [of the team/organization] in terms of our resource” (Community Leisure Sport UK, Scotland, 163).

Official regulations, guidelines, and charters on the appreciation of diversity, diversity management, and prevention of violence are supposed to be beneficial. The Austrian Sport Organization stressed the importance of clearly naming the dimensions of diversity, such as sexual orientation, in the official guidelines and regulations. Furthermore, the implementation of the topic on diversity and inclusion of sexual minorities in the educational structures of sport—for coaches, officials, referees, employees, board members and so on—is seen in a positive light. To justify to higher management levels and potential funding bodies the need for inclusive strategies and measures and to draw a comprehensive picture of the situation of LGBT+ in sports, many organizations stress the relevance of empirical facts and figures.

On the micro level, the organizations are highly aware of the significance of using sensitive and inclusive language in the context of discrimination in sports. People using homo- and transnegative language are often not aware that it might be perceived as discriminatory by LGBT+ individuals. Instead, the word “gay” is commonly used in sport contexts to describe something in a negative way, but not with the intention to harm LGBT+ individuals. Raising awareness for the discriminatory effect of homo- and transnegative language in sports is an important concern of the organizations. “And I think particularly with LGBTI [inclusion], you want to have language that is inclusive (…) because it does, it seems to change quite a lot” (Scottish Disability Sport, 216–217).

Apart from that, it became obvious that the organizational engagement with the topic of diversity and inclusion often depends on highly dedicated individuals within the organizations (not necessarily at the leadership level), who are committed to advance the issue, implement initiatives and measures, involve relevant stakeholders, and cooperate with them.

## Discussion

Against the background of growing empirical research on LGBT+ people in sports from Anglophone countries, this paper enhances the research body on both the experiences of LGBT+ people in organized European sports in general and the various challenges with regard to gender identity in particular. Aside from that, the combination of quantitative findings on the experiences of LGBT+ athletes and qualitative findings on organizational strategies for diversity and inclusion represents a major contribution of this study.

The current findings indicate fairly equal sports participation rates of cisgender and non-cisgender individuals in Europe (Kulick et al., 2018). Transgender males, being the most active group, reflect structural conditions of the sport system. Compared to transgender females, transgender males challenge the level playing field less strongly because they are not accused of unfair physical advantages. Similarly, transgender males challenge the level playing field less than non-binary athletes because the former fit into the sex binary (Fink, 2008; Griffin, 2012; Lucas-Carr and Krane, 2012). These perceptions culminate in fewer barriers for and fewer prejudices against transgender males in the domain of sport, which in turn increase transgender males' participation (Lucas-Carr and Krane, 2012).

LGBT+ athletes in organized sports contexts in Europe perceive transnegativity as a major problem in sports and even as a bigger problem than the Scottish respondents perceived in the studies by Smith and colleagues some years ago (2012a,b). National differences in the prevalence of transnegativity amongst European countries, as indicated in the overall OUTSPORT results (Menzel et al., 2019), as well as negative changes over time might have contributed to this difference. Within the group of non-cisgender respondents, those who reject the binary assess the problem to be the biggest, reflecting the sex-segregation in sports, which is an even higher barrier for non-binary athletes than for male or female transgender athletes.

Despite the generally high awareness of transnegativity, the share of respondents with personal negative experiences is relatively low, compared to findings by Demers (2017) or Smith et al. (2012a). Different periods considered may partly account for the differences: while the current study looked at incidents in the last 12 months, Demers (2017) and Smith et al. (2012a) focused on lifetime experiences. Apart from methodological issues, the spread of more inclusive sporting realities for LGBT+ individuals (Travers and Deri, 2010) and self-exclusion processes based on proximal stressors serve as arguments for reduced transnegative episodes in sport. Elling-Machartzki (2015, p. 9) and Hargie et al. (2017) stress that expectations of being harassed or discriminated in sport contexts and the “fear of the felt stigma” that is “related to transgenderism” led to self-exclusion and thus to LGBT+ sporting populations that are less vulnerable to harassment and discrimination.

Within the group of LGBT+ athletes, non-cisgender athletes are identified as the most vulnerable group in organized sports in general and particularly with regard to structural discrimination (i.e., facing unequal opportunities, unfair treatment, or exclusion) (Jones et al., 2017b). Non-cisgender athletes challenge the sex binary and sex-segregated sport systems and the alignment to either male or female teams that is required for participation in competitive structures (Griffin, 2012; Krane et al., 2012; Hartmann-Tews et al., in press). Even LGBT+ specific settings seem to be less safe for non-cisgender than for cisgender athletes (Travers and Deri, 2010; Caudwell, 2014; Semerjian, 2019), which emphasizes the importance of trans-only sporting environments as an external facilitator for sport participation of transgender individuals (Jones et al., 2017a). Transgender athletes' negative experiences are reflected in calls, particularly from non-cisgender athletes, for diversity training and more inclusive policies. The disadvantages of non-cisgender athletes are also evident in the different status of transgender athletes' issues, lagging behind diversity strategies and inclusion of lesbian, gay and bisexual individuals, as the interviews indicated. But the will “to overcome resistance to change” (Cunningham, 2007, p. 306) is evident in many sports organizations, which stress the need for inclusive policies instead of the case-by-case decisions currently being taken.

Concepts of hegemonic masculinity and heteronormativity have been identified as important determinants for LGBT+ discrimination. Verbal abuse, the most common form of discrimination (particularly for cisgender athletes) is mainly anchored in expectations of heteronormative, male hegemony, which strongly devalues homosexual orientations (Kossakowski et al., 2020). Moreover, the impact of performance level on discrimination experiences of LGBT+ athletes is closely related to the importance of hegemonic masculinity in competitive sport, which is more strongly anchored in higher performance levels than in recreational sports settings (Bush et al., 2012; Cunningham, 2012; MacDonald, 2018). Individuals who are embedded and socialized in sporting cultures identify more strongly as athletes, particularly if they are engaged in competitive sports, and therefore express more sexual prejudices than athletes with a lower athletic identity (Bush et al., 2012; Cunningham, 2012).

The high prevalence of verbal abuse and severe physical incidents supports findings from Demers (2017) and Smith et al. (2012a), but the pervasiveness of incidents related to physically crossing lines (i.e., being shoved, pushed, or inappropriately touched) is an alarming sign for the sport culture. Referring to gender identity, being looked at in a derogatory way, or being misgendered happen more often to transgender and gender non-conforming athletes (Travers and Deri, 2010; Devís-Devís et al., 2018; Semerjian, 2019). The interviewed organizations recognize these problems and emphasize the need to raise awareness and knowledge on the importance of a sensitive language in sports contexts and perceive the integration of LGBT+ (particularly transgender issues) into educational structures as an important measure. The need for this is strengthened by two aspects: first, the finding that coaches discriminate against non-cisgender athletes more often than cisgender athletes; and second, academic agreement on coaches playing a major role in raising awareness, establishing an inclusive climate, and addressing problems concerning correct pronouns and the changing room situation (Rulofs, 2012; Morris and Van Raalte, 2016; Demers, 2017). Implementing the topic into educational structures and offering diversity training are not only perceived as important measures to tackle transnegativity by the organizations but also by the respondents themselves. Additionally, non-cisgender athletes express the need for more awareness and acceptance and the need for gender-neutral changing rooms, with changing rooms having been consistently identified as very unsafe spaces (e.g., Lucas-Carr and Krane, 2012; Smith et al., 2012a; Elling-Machartzki, 2015; Jones et al., 2017a).

## Conclusion

Before concluding thoughts will be presented, some limitations to the studies and future perspectives have to be mentioned.

First, it should be noted that the generalizability of the study's results is limited, since the present sample consists of self-selected respondents from an international population with unknown socio-structural parameters. Second, the questionnaire was only available in five languages, leading to a considerable percentage of the European respondents who could not respond in their mother tongue. This might have resulted in problems in understanding the questions and therefore in biased results and/or a biased sample, accounting for the rather high education level of the respondents. Third, although the sample size is satisfactory, the subsamples for non-cisgender athletes with negative experiences in specific contexts is sometimes insufficient to draw reliable conclusions. Additional quantitative research on transgender and non-conforming athletes is needed to further examine some discriminatory experiences that have been indicated in the current study, such as LGBT+ settings as unsafe spaces for non-cisgender athletes (Travers and Deri, 2010; Tagg, 2012; Caudwell, 2014). Fourth, the quantitative approach is unable to reflect the manifold, diverse, and different individual experiences of LGBT+ athletes in various sports contexts, settings, countries, and so on (Semerjian and Cohen, 2006). But it is able to provide comparable insights into the experiences of LGBT+ athletes with regard to different dimensions such as gender identity, performance levels, or LGBTI+ reference of the sport setting. Thus, the necessary reduction of complexity in quantitative research highlights the need for further research and, preferably, mixed-method approaches. Fifth, the qualitative study is limited by some methodological issues in conducting the interviews, which complicated drawing differentiated and comprehensive pictures in all project partner countries. Moreover, due to the broader focus on LGBT+ inclusion and the rather elementary stages in non-cisgender inclusion of sports organizations in all countries, transgender policies could not have been adequately discussed. Integrating more countries in future research and providing scientifically based support for the inclusion of transgender individuals in sports organizations (especially competitive structures) might be beneficial.

The aim of the paper was to shed light on the often neglected inconsistencies between LGBT+ athletes in sports in Europe. Our findings strikingly support the necessity of disbanding the LGBT+ umbrella, as lesbian, gay, bisexual, transgender, and non-binary athletes are facing different challenges and different proximal and distal stressors in participating in organized sports contexts. Two realities seem to account for the different sporting realities of cisgender and non-cisgender athletes: first, transgender athletes thoroughly challenge people's assumptions of distinct gender binaries and heteronormativity, and second, they challenge the gender boundaries of the sex-segregated (competitive) sports system. Transgender participation in sport “belies the myth of the level playing field and the myth of gender binary on which it rests” (Griffin, 2012, p. 100). Instead of discussing the implied unfair physical advantages of transgender (female) athletes, Lucas-Carr and Krane (2011, in Semerjian, 2019, p. 151) call for a shift to discuss the “trans disadvantage” due to negative experiences and ongoing challenges in sports that complicate sports participation, practice routines, competition, and so on. Together with the positive outcomes of being physically active, this strengthens the need for establishing welcoming organizational cultures and inclusive structures enabling transgender athletes to participate in organized sports settings without the fear of being verbally insulted, misgendered, stigmatized, or discriminated against. These calls are being heard in sports organizations but are not yet sufficiently implemented as the interviews with the sport organizations indicate. The study emphasizes that change is needed on macro, meso, and micro levels (Cunningham, 2012): first and foremost, to raise awareness for transgender issues and implement inclusive policies that allow transgender athletes “to compete as they wish” (Semerjian, 2019, p. 159); secondly, to bring the coaches in to establish welcoming and safe sporting realities; and lastly, to provide gender-neutral changing/locker rooms in sports facilities and promote sensitive and inclusive language.

## Data Availability Statement

The datasets presented in this article are not readily available because data is part of a funded Project by EU Commission.

## Ethics Statement

The studies involving human participants were reviewed and approved by Ethics commitee of the German Sport University Cologne. Written informed consent from the participants' legal guardian/next of kin was not required to participate in this study in accordance with the national legislation and the institutional requirements.

## Author Contributions

BB: head of the manuscript: first draft of the structure and content of the manuscript, writing everything except for methods and results, and finalizing and submitting the manuscript. TM: involved in the planing process, wrtiting the sextions on methods and results, and feedback on the final manuscript. IH-T: involved in the planing process and data selection, and feedback on the final manuscript. BB, TM, and IH-T: members of the OUTSPORT Project Team of the German Sport University. All authors contributed to the article and approved the submitted version.

## Conflict of Interest

The authors declare that the research was conducted in the absence of any commercial or financial relationships that could be construed as a potential conflict of interest.

## Footnotes

^1^As part of the project OUTSPORT the research was funded by ERASMUS+. The aim of OUTSPORT was to develop innovative and educational approaches to prevent violence and tackle discrimination in sports, based on sexual orientation and gender identity. The European Commission support for the production of this publication does not constitute an endorsement of the contents which reflects the views only of the authors, and the Commission cannot be held responsible for any use which may be made of the information contained therein.

^2^The term “non-cisgender individuals” is used as a catchall term for transgender women, transgender men, non-binary and non-identifying individuals, and thus goes beyond the umbrella term “transgender.” It is not intended to denote otherness or deviation.

^3^Respondents had to be at least 16 years old to participate in the survey. At the time of respondents' birth or earlier, none of the EU member states had officially recognized a third sex at birth.

^4^From all 2,282 cases, 7.5% chose the option “don't know,” and 0.1% did not respond.

^5^Due to an unintended permeability in the filter guidance, respondents who had not had negative experiences in the last 12 months also answered this question. As perceptions about helpful actions to tackle discrimination are probably (mostly) independent from immediate experiences of discrimination and for the sake of a higher number of cases, the table includes all active sports participants in organized sports who made a valid statement in this question (*n* = 733).
